# Peptidoglycan permease AmpG1 couples lytic transglycosylase activity to muropeptide import in *Acinetobacter baumannii*

**DOI:** 10.1016/j.jbc.2026.113362

**Published:** 2026-07-24

**Authors:** Taeyeong Kim, Reagan Lee, Yerim Park, Jihye Bae, Jihyeon Min, Woojun Park

**Affiliations:** Laboratory of Molecular Environmental Microbiology, Department of Environmental Science and Ecological Engineering, Korea University, Seoul, Republic of Korea

**Keywords:** Gram-negative bacteria, cell wall, peptidoglycan, muropeptide transport, protein–protein interaction, bacterial metabolism

## Abstract

Peptidoglycan (PG) recycling contributes to envelope integrity and antibiotic resistance in gram-negative bacteria. Many species import PG turnover products through multiple systems, including AmpG, OppBCDF, MurP, and NagE. *Acinetobacter baumannii*, however, lacks OppBCDF, MurP, and NagE but encodes three *ampG* homologs, suggesting a particular reliance on AmpG-mediated recycling. Bioinformatic analysis identified one homolog, AmpG1, which harbors a distinctive periplasmic domain conserved within the *Acinetobacter* genus. Because PG turnover occurs in the periplasm through the action of PG hydrolases, we used AlphaFold 3-based prediction to identify potential interacting partners of AmpG1 and found the soluble lytic transglycosylase (Slt) as a top candidate. Microscale thermophoresis confirmed direct binding between Slt and the periplasmic domain of AmpG1. LC-MS/MS analysis of cellular extracts revealed that Δ*ampG1* and Δ*ampG1*Δ*slt* mutant cells accumulated GlcNAc-1,6-anhydroMurNAc-tri/tetrapeptides and exhibited reduced levels of UDP-MurNAc-pentapeptide, changes not observed in Δ*slt* mutant cells. Moreover, Δ*ampG1* mutant cells showed aberrant morphology and increased β-lactam sensitivity. This sensitivity results from disrupted PG metabolism due to loss of AmpG1-dependent muropeptide import, rather than from altered β-lactamase activity or membrane permeability. Together, these results support a model in which the periplasmic domain of AmpG1 directly interacts with Slt to couple glycan cleavage with import, thereby promoting efficient uptake of anhydromuropeptides and sustaining precursor supply. These findings delineate a key step in the PG recycling pathway of *A. baumannii*.

Unlike gram-positive bacteria with an exposed cell wall, gram-negative species remodel their peptidoglycan (PG) within the periplasm enclosed by dual membranes. This compartmentalization enables efficient retention and reuse of turnover fragments, while gram-positive bacteria lose these materials to the environment and depend largely on *de novo* cell wall synthesis (1, 2). PG remodeling poses a fundamental challenge, as the rigid cell wall must be locally cleaved by autolytic hydrolases such as endopeptidases, amidases, and lytic transglycosylases to permit expansion, while new glycan strands are concurrently synthesized by transglycosylases and crosslinked by transpeptidases (penicillin-binding proteins) to preserve mechanical integrity and prevent lysis (3). To maintain this delicate balance between degradation and synthesis, bacteria utilize PG recycling pathways that capture turnover-derived fragments and channel them back into precursor biosynthesis, improving both structural homeostasis and metabolic efficiency. *De novo* synthesis of the UDP-MurNAc-pentapeptide (UDP-M5) precursor is energetically demanding, involving at least four ATP-dependent ligase reactions catalyzed by MurC, MurD, MurE, and MurF. Consequently, efficient PG recycling provides a major energetic advantage by reducing the burden of continual precursor regeneration (4). In contrast, the primary recycling route salvages the key tripeptide (L-Ala–D-Glu–meso-DAP) and reattaches it to UDP-MurNAc through the murein peptide ligase (Mpl), consuming only a single ATP and achieving an estimated ≥80% reduction in ATP expenditure for this step (5). In the well-studied model *Escherichia coli*, this recycling network is highly optimized, with muropeptides imported and processed by the AmpG permease together with AmpD and NagZ (6, 7, 8). The PG sacculus undergoes rapid turnover, with approximately 50% of its material degraded each generation, and more than 90% of the resulting fragments are efficiently recovered (9, 10, 11). The remaining ∼10% that escape recycling are released into the extracellular milieu (12).

Although the canonical *E. coli* pathway provides a foundational model, comparative genomics reveals remarkable diversity in how bacteria organize and regulate PG recycling and turnover systems (13). For example, *E. coli* encodes eight lytic transglycosylases (LTs) and a complete set of importers, including OppBCDF, MurP, and NagE, whereas *A. baumannii* carries only six LTs and lacks the Opp, MurP, and NagE systems entirely, positioning AmpG as the indispensable transporter for its recycling pathway. By contrast, *Pseudomonas aeruginosa* possesses 11 LTs. Moreover, certain pathogens have evolved lineage-specific enzymes, such as PBP_sal_ in *Salmonella* and ElsL in *Acinetobacter baumannii*, that are absent from *E. coli* (14). In the canonical gram-negative model, the inner membrane permease AmpG acts as a crucial conduit, retrieving anhydromuropeptides generated by LTs during cell wall turnover and transporting them into the cytoplasm for processing by NagZ and AmpD and subsequent recycling into new precursors (6, 15). A striking example is *Neisseria gonorrhoeae*, a pathogen with rapid PG turnover but an inefficient AmpG permease that releases ∼15% of PG monomers as toxic, proinflammatory fragments (16). This defect was corrected by replacing *ampG* with the efficient meningococcal ortholog, which successfully limited the release to approximately 4% (16). This deliberately reduced recycling efficiency in *N. gonorrhoeae* represents an important pathogenic adaptation that promotes the release of abundant, proinflammatory PG monomers, thereby exacerbating host inflammatory responses (16, 17). Collectively, these variations underscore that PG recycling is not a uniform or conserved process but rather a highly adaptable system that has been diversified across bacterial lineages. Its components and efficiency have been evolutionarily tailored to distinct ecological and pathogenic contexts, ranging from metabolic optimization in commensals such as *E. coli* to host-modulating strategies in pathogens like *N. gonorrhoeae*.

The role of AmpG as the sole muropeptide importer in *A. baumannii* makes it critically important for the cell recycling pathway. PG turnover occurs within a narrow periplasmic space, directly measured by cryo-electron microscopy to be only ∼21 to 24 nm wide in nondeformed regions (18). This compartment is densely crowded with periplasmic proteins and the PG sacculus itself, generating a physically congested environment that limits diffusion efficiency. Consequently, muropeptide fragments generated by LTs must traverse this ∼20 nm gap to locate an AmpG transporter before diffusing away. The genomic context of this import process in *A. baumannii* is also unusual, as the genome encodes three distinct *ampG* homologs (19). Structural modeling and sequence analyses reveal that one of these contains a unique periplasmic insertion domain of ∼196 amino acids, absent from the others (20). This unique domain architecture suggested several potential functions essential for solving the spatial challenge of import, such as serving as a periplasmic anchor to localize the permease at sites of active PG turnover or functioning as a protein–protein interaction scaffold to directly recruit specific PG hydrolases and channel muropeptides. Using a combined molecular genetic and biochemical approach with phylogenetics and structural predictions, we tested these hypotheses and identified an interaction between this specific domain and the soluble lytic transglycosylase (Slt).

## Results

### Functional dominance and unique structural features of AmpG1

**Figure 1:**
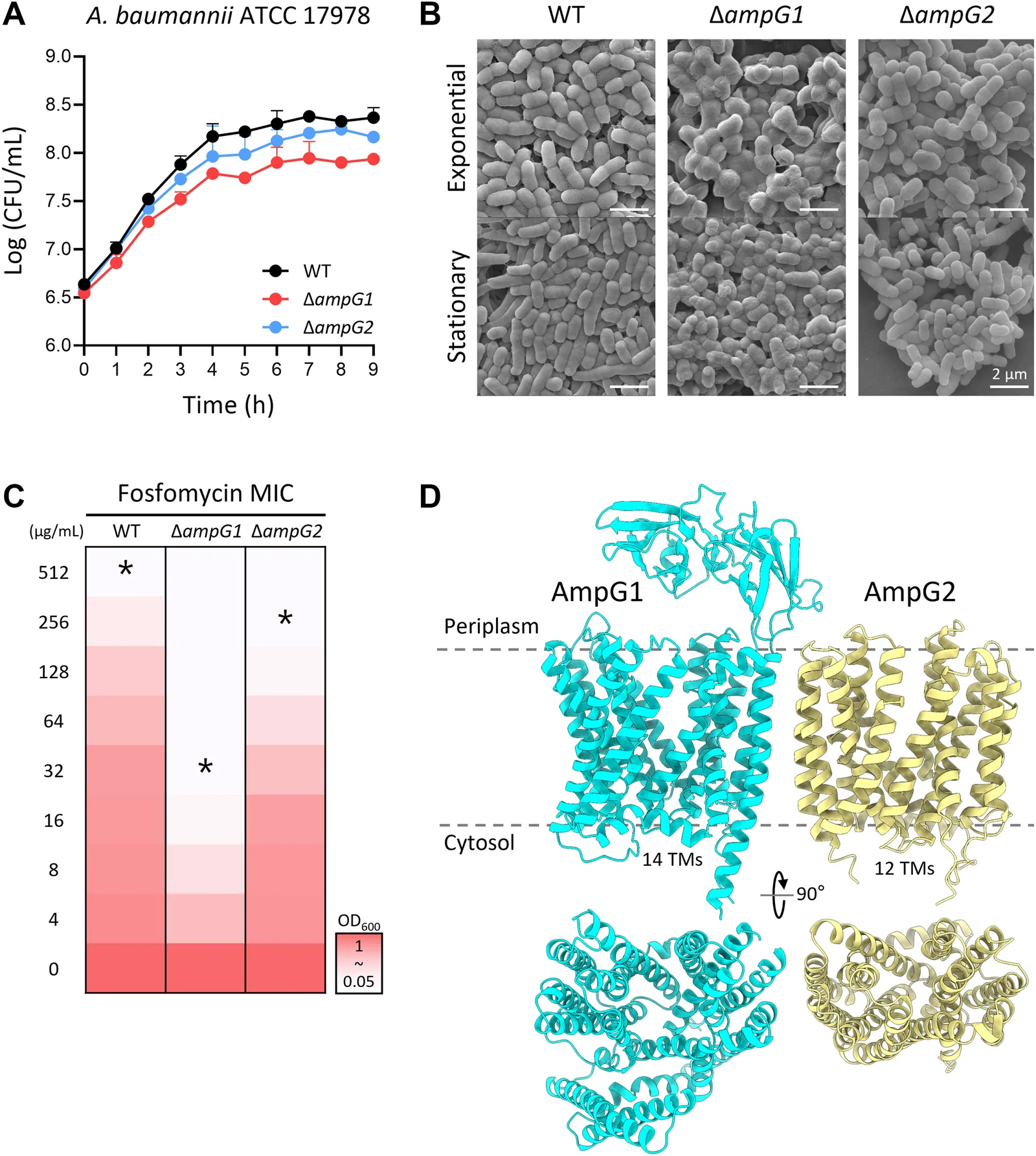
**AmpG1 as the primary permease in *A. baumannii.****A*, growth curves of WT, Δ*ampG1*, and Δ*ampG2* in *A. baumannii* ATCC 17978. Data show mean ± s.d. based on n = 3 biological replicates. At time points with no visible bars, the s.d. is smaller than the symbol. *B*, SEM analysis of WT, Δ*ampG1*, and Δ*ampG2* acquired in exponential and stationary phases. Scale bars, 2 μm. *C*, fosfomycin susceptibility by broth microdilution. *A*_600_ was measured at 24 h. Left labels indicate fosfomycin concentration in μg/ml. Asterisks indicate the MIC for each strain, defined as the lowest fosfomycin concentration at which no growth was detected. *D*, AlphaFold 3-predicted structures of AmpG1 and AmpG2. pTM scores are 0.86 for AmpG1 and 0.88 for AmpG2. MIC, minimum inhibitory concentration.

*A. baumannii* American Type Culture Collection (ATCC) 17978 encodes three *ampG* homologs that share modest amino acid sequence identity with *E. coli* AmpG (AmpG1, 30.0%; AmpG2, 24.6%; AmpG3, 28.1%). We prioritized *ampG1* and *ampG2* for functional analysis because deletion of *ampG3* caused no discernible morphological defect, suggesting a minor or redundant role (19). Using CRISPR-Cas9 mutagenesis, Δ*ampG1* and Δ*ampG2* mutant strains were constructed, both of which exhibited growth inhibition in LB medium relative to the wildtype (WT), with a more pronounced defect in Δ*ampG1* mutant cells (Figs. 1*A* and S1). These observations indicate that *A. baumannii* relies on AmpG-mediated import for normal growth, unlike *E. coli*, in which alternative uptake routes compensate for *ampG* loss (21). Scanning electron microscopy (SEM) analysis revealed that while Δ*ampG2* mutant cells retained a WT-like morphology across growth phases, Δ*ampG1* mutant cells exhibited morphological defects that were already apparent during the exponential phase and became significantly more severe and pronounced during the stationary phase, displaying extensive envelope deformation and bulging. (Fig. 1*B*). This functional divergence demonstrates that AmpG1 and AmpG2, despite being homologs, are not interchangeable and have likely evolved for distinct, specialized roles within cell wall metabolism. Complementation with *ampG1* restored normal morphology (Fig. S2), confirming that *ampG1* is the principal homolog required for cell-shape maintenance. To further assess the physiological consequence of AmpG loss, the *de novo* synthesis enzyme MurA was inhibited using fosfomycin. Hypersusceptibility to this drug is a specific marker for defective PG recycling, as the pathway is no longer able to compensate for the inhibited *de novo* synthesis (22, 23, 24, 25). Because *A. baumannii* lacks alternative muropeptide transporters, disruption of AmpG-mediated recycling forces the cell to rely predominantly on *de novo* synthesis to supply its PG precursors. Consistent with this prediction, the Δ*ampG1* mutant cells displayed a 16-fold reduction in the fosfomycin minimum inhibitory concentration (MIC), dropping from a WT value of 512 μg mL^−1^ to 32 μg mL^−1^, whereas the Δ*ampG2* mutant cells remained comparable to WT at 256 μg mL^−1^ (Fig. 1*C*). Collectively, these results establish the nonredundant functions of the homologs, identifying AmpG1 as the dominant permease for PG recycling while implying a distinct physiological role for AmpG2. We also assessed whether simultaneous loss of AmpG homologs compromises cell viability. The Δ*ampG1*Δ*ampG2* double mutant was viable under the tested laboratory growth conditions and recovered growth to near-WT levels rather than showing lethality (Fig. S3*A*). This strain showed increased β-lactam MICs, partial recovery of lysozyme and fosfomycin tolerance relative to Δ*ampG1* mutant cells, and marked EDTA hypersensitivity (Fig. S3, *B*–*E*). These data suggest that simultaneous loss of AmpG1 and AmpG2 induces an altered envelope state rather than cell death.

To investigate the structural basis for this functional divergence, the three-dimensional structures of AmpG1 and AmpG2 were first predicted using AlphaFold 3. Comparative topology analysis highlighted the resulting structural divergence between the two permeases, with AmpG1 (729 aa) containing 14 transmembrane (TM) helices while AmpG2 (407 aa) possesses 12 (Fig. 1*D*). AmpG belongs to the major facilitator superfamily (MFS), whose members typically comprise 12 to 14 TMs. While the 14-TM core of AmpG1 is consistent with known AmpG architecture (20), the AlphaFold 3 model indicated that AmpG1 also possesses a unique domain, located in the middle of the protein sequence, that is absent from AmpG2 and other canonical MFS transporters. Topology predictions using PPM 2.0 and Protter consistently placed this domain on the periplasmic side of the inner membrane (Fig. S4, *A* and *B*), and we subsequently refer to it as the periplasmic domain (PPD). The AlphaFold 3 model of AmpG1 shows that PPD (∼196 aa) is connected to the main transporter body, which consists of 14 TMs and a bundle bridge (∼523 aa), through a short linker region (∼10 aa) (Fig. S4*A*). A color-coded topology map clarifies the architecture by grouping TM1 to TM6 as the N-bundle and TM9 to TM14 as the C-bundle and linking them through TM7 and TM8 with a red bundle bridge while highlighting the green PPD as a large insertion between TM11 and TM12 (Fig. S4*B*). A Hidden Markov model (HMM) search subsequently identified this domain as belonging to the Ig-like fold family and revealed its restriction to the *Acinetobacter* genus, indicating a genus-specific adaptation rather than a conserved AmpG feature (Fig. S4*C*). Given the limited PG-recycling transport capacity of *A. baumannii*, the coexistence of three *ampG* homologs likely represents compensatory evolution, with AmpG1 serving as the principal anhydromuropeptide permease distinguished by its 14 TMs and genus-specific PPD.

### Altered PG composition and mislocalized PG insertion in ΔampG1 mutant cells

**Figure 2:**
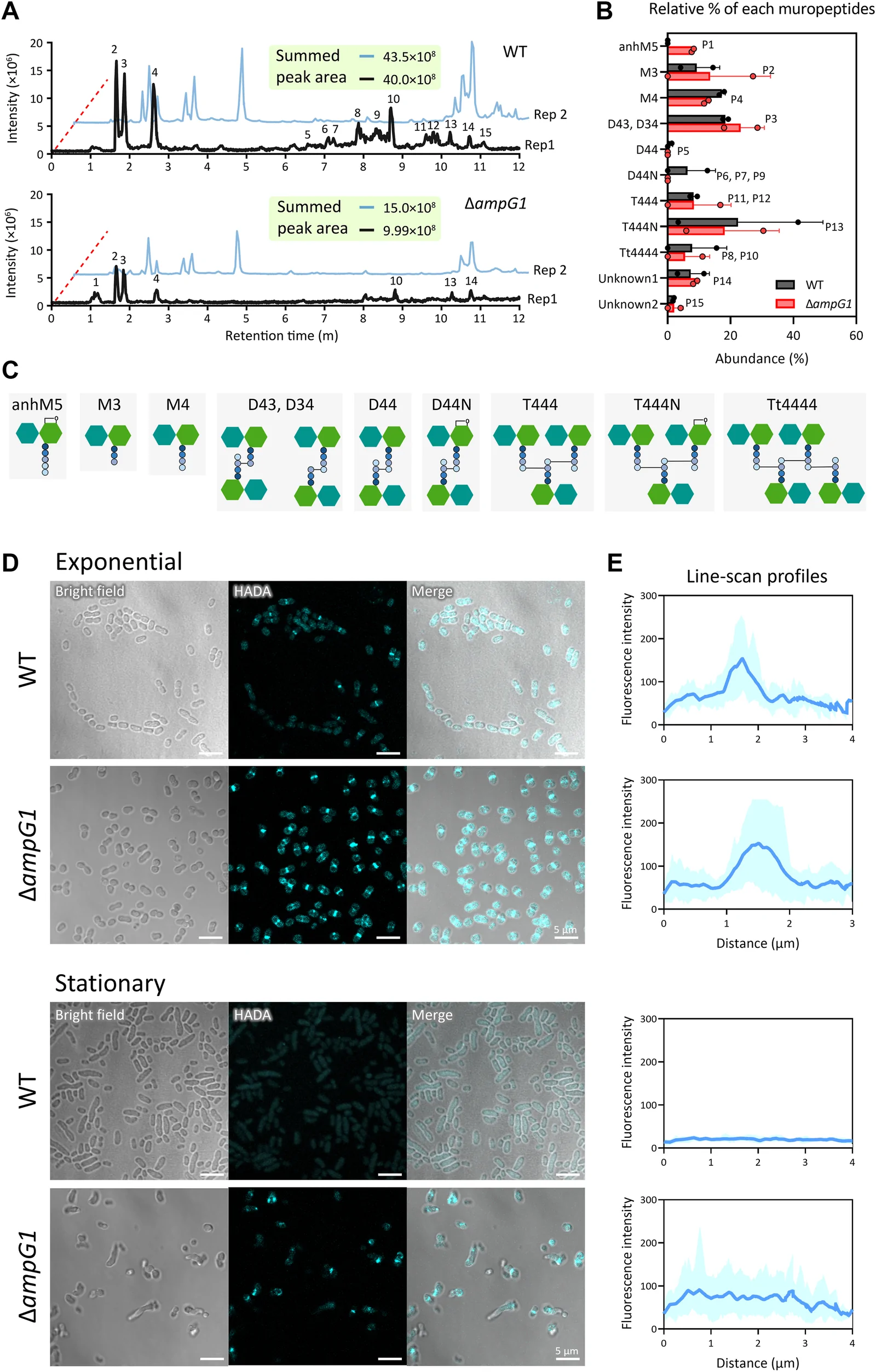
**Altered PG composition and mislocalized PG insertion in Δ*ampG1* mutant cells.***A*, LC-MS/MS chromatographic profiles of muropeptides released from lysozyme-digested PG sacculi isolated from stationary-phase WT and Δ*ampG1* mutant cells in two independent biological replicates. Rep. 1 and Rep. 2 are shown in *black* and *blue*, respectively, and the Rep. 2 traces are vertically offset for clarity. Values in the boxes indicate the summed integrated peak area for each replicate. Numbered peaks correspond to the peak identifiers associated with the muropeptide species in *panel B* and to the replicate-specific assignments listed in Fig. S5. *B*, relative abundance of the indicated muropeptide species in WT and Δ*ampG1* mutant cells from two independent biological replicates. Individual points represent biological replicates, and bars show the mean values for descriptive presentation. Peak identifiers P1–P15 correspond to peaks 1 to 15 in *panel A*. Multiple peak identifiers associated with one muropeptide indicate chromatographically resolved peaks assigned to the same species. Complete replicate-specific retention times, observed m/z values, and relative-abundance data are provided in Fig. S5. *C*, schematic structures of the assigned muropeptide species. *D*, Confocal laser scanning microscopy image analysis of HADA fluorescence in WT and Δ*ampG1* mutant cells during exponential and stationary phases. Scale bars, 5 μm. *E*, Line-scan fluorescence profiles for WT and Δ*ampG1*. PG, peptidoglycan.

Bacterial cell shape is largely dictated by the PG sacculus, and perturbations in PG-modifying enzymes that sculpt this structure typically result in severe morphological abnormalities (26, 27). Consistent with this principle, scanning electron microscopy revealed pronounced shape defects in stationary phase Δ*ampG1* mutant cells (Fig. 1*B*). To determine whether these morphological changes reflected underlying alterations in sacculus composition, LC-MS/MS analysis was performed on muropeptides released by lysozyme digestion from the insoluble PG sacculus isolated from this stage, reflecting the composition of the cross-linked glycan strands rather than soluble turnover products (Figs. 2, *A*–*C* and S5). The summed muropeptide peak area in Δ*ampG1* mutant cells (15.0 × 10^8^ and 9.99 × 10^8^) was substantially lower than in WT (43.5 × 10^8^ and 40.0 × 10^8^), indicating a reduction in overall PG content (Fig. 2*A*). Despite this overall decline, the 1,6-anhydroMurNAc-pentapeptide, a muropeptide carrying the 1,6-anhydro ring that marks the terminus of a glycan strand cleaved by lytic transglycosylases, was consistently detected in the Δ*ampG1* sacculus, where it accounted for approximately 8% of the total muropeptide pool, yet it remained below the limit of detection in WT cells (Fig. 2, *B* and *C*) (3). Because 1,6-anhydroMurNAc-pentapeptide retains both the 1,6-anhydro cap that terminates a glycan strand and the full, unprocessed pentapeptide stem, its appearance in the Δ*ampG1* wall reflects an accumulation of glycan strand termini that are generated by lytic transglycosylase cleavage but are not carried forward through the processing steps that normally consume them during sacculus maturation. Together, these changes demonstrate that the loss of AmpG1 reduces overall PG content and reshapes the stationary phase cell wall toward unprocessed glycan-strand ends, providing a structural basis for envelope deformation and directly linking the mutant's morphological abnormalities to defined alterations in the chemical building blocks of its sacculus.

Because local defects in PG architecture can misdirect cell-wall growth, transpeptidase activity was next mapped across growth phases using the fluorescent D-amino acid probe 7-hydroxycoumarin-3-carboxylic acid-3-amino-D-alanine (HADA), which labels sites of active PG synthesis (Fig. 2*D*) (28, 29). During exponential growth, both WT and Δ*ampG1* mutant cells exhibited septal HADA enrichment, confirmed by line-scan profiles showing sharp intensity peaks at mid-cell (Fig. 2, *D* and *E*, top). In the stationary phase, WT displayed faint, diffuse labeling consistent with low PG synthesis, whereas Δ*ampG1* mutant cells showed intense, aberrant signals along bulged and irregular membrane regions. Corresponding line-scans broadened and shifted away from the septum (Fig. 2, *D* and *E*, bottom), indicating mislocalized PG insertion at deformed sites. To test whether this spatial misregulation was accompanied by altered transpeptidase abundance, active PBPs were quantified using Bocillin labeling. The visualization of the resulting protein gel showed that major PBPs (PBP1a, 1b, 2, and 3) had the highest band intensity, which appears as the darkest fluorescence on the image (Fig. S6), in Δ*ampG1* mutant cells than in WT, confirming a compensatory upregulation in the amount of active enzyme. Despite this compensatory upregulation, the Δ*ampG1* retained their aberrant morphology and their mislocalized pattern of synthesis, indicating that envelope instability arises not from insufficient enzyme levels but from limited precursor supply and spatial misalignment of synthesis. Collectively, these findings show that loss of AmpG1 decouples precursor recycling from spatial control of PG assembly, leading to mislocalized cell-wall insertion, aberrant PBP activation, and compromised envelope homeostasis during stationary phase.

### Direct interaction between Slt and the PPD of AmpG1

**Figure 3:**
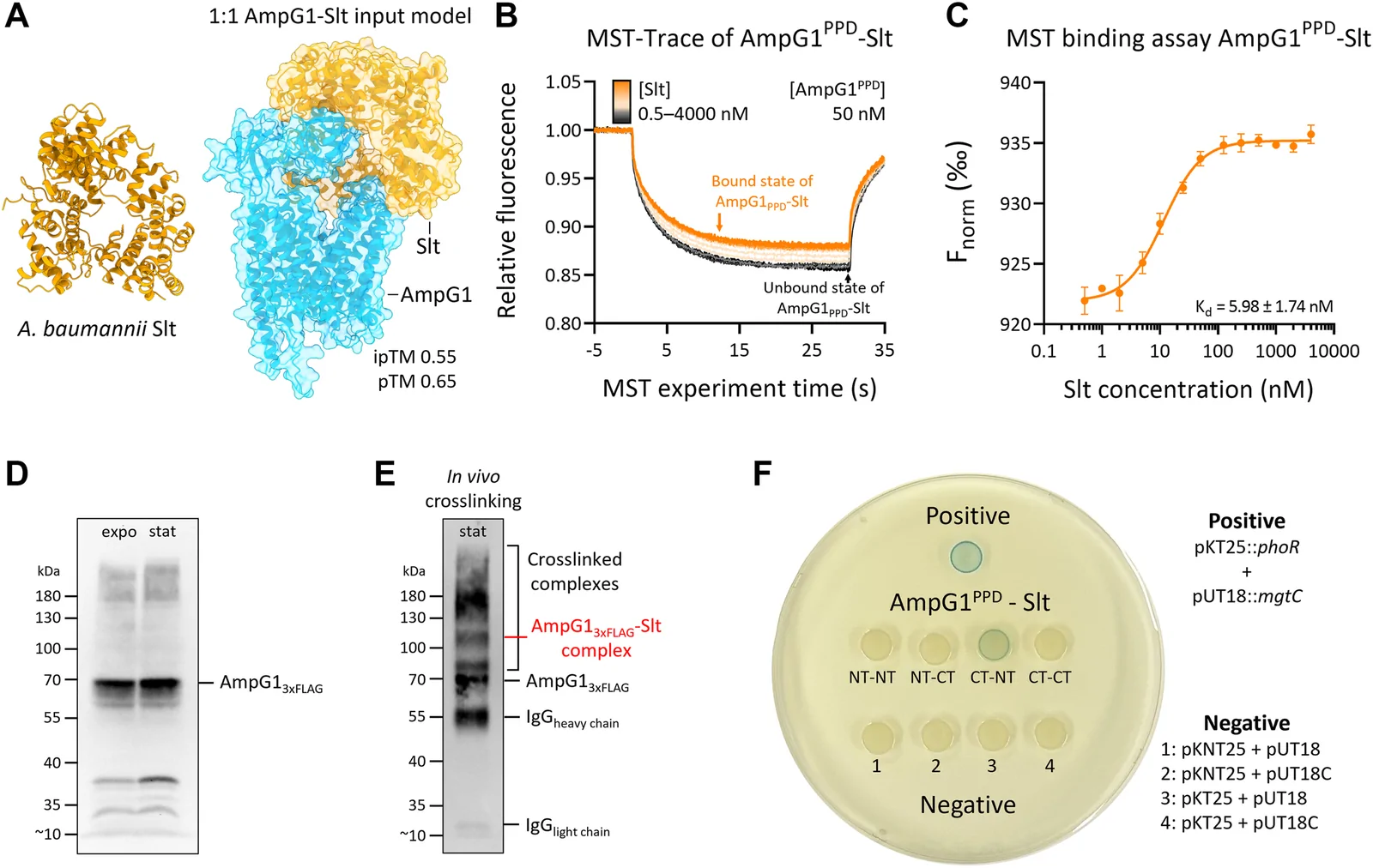
**Confirmation of direct AmpG1-Slt binding by *in vitro* and *in vivo* analyses.***A*, AlphaFold 3-predicted AmpG1–Slt complex generated using one AmpG1 chain and one Slt chain lacking its N-terminal signal peptide, corresponding to a 1:1 input stoichiometry. The isolated signal peptide-deleted Slt structure is shown at left for clarity. Confidence scores were ipTM 0.55 and pTM 0.65. *B*, representative MST traces obtained with 50 nM fluorescently labeled AmpG1^PPD^ and increasing concentrations of Slt ranging from 0.5 to 4000 nM. *C*, MST binding isotherm for the AmpG1^PPD^–Slt interaction fitted using a standard one-site binding model, yielding an apparent K_d_ of 5.98 ± 1.74 nM. *D*, anti-FLAG immunoblot of AmpG1_3xFLAG_ in exponential and stationary phase samples showing a major band migrating at approximately 70 kDa. *E, in vivo* chemical crosslinking followed by anti-FLAG immunoprecipitation of AmpG1_3xFLAG_ from stationary phase cells reveals additional high-molecular-mass species under crosslinking conditions, and Slt was detected in the crosslinked immunoprecipitate by LC-MS-based proteomics. *F,* adenylate cyclase-based bacterial two-hybrid assay testing AmpG1^PPD^ and Slt. AmpG1^PPD^ was cloned into the T25 fusion vectors pKNT25 and pKT25, and Slt was cloned into the T18 fusion vectors pUT18 and pUT18C, enabling N-terminal and C-terminal fusion orientations. *Blue* coloration indicates interaction. Positive control was pKT25::*phoR* with pUT18::*mgtC*. Negative controls were empty vector combinations pKNT25 with pUT18, pKNT25 with pUT18C, pKT25 with pUT18, and pKT25 with pUT18C. MST, microscale thermophoresis; Slt, soluble lytic transglycosylase.

Given the functional dominance of AmpG1 and its unique PPD, the specific role of this domain was next investigated. A HMM search identified this domain as belonging to the Ig-like fold family (Fig. S4*C*), a structural motif that has been widely adopted in evolution precisely because it is “particularly good for mediating interactions” (30). Based on this well-established characteristic, it was reasoned that the PPD likely serves as a scaffold to recruit its own interacting partner to facilitate the function of the AmpG1 transporter. Since PG turnover by hydrolases occurs in the periplasm, where this PPD also resides, we hypothesized that the PPD acts to recruit a periplasmic partner, thereby directly coupling glycan cleavage to transport. Following this reasoning, a list of 20 periplasmic enzymes associated with PG biosynthesis or recycling was compiled to screen as candidate partners. Complexes between the PPD and each enzyme were then predicted using AlphaFold 3, evaluating interaction confidence using ipTM and pTM (Table 1). While this screen included various relevant enzymes such as PBPs and other peptidases, Slt showed the highest interaction confidence scores (ipTM 0.55; pTM 0.65), and its complex model predicted direct binding to the PPD (Fig. 3*A*). This finding is highly compelling, as Slt is the precise lytic transglycosylase that cleaves PG glycan strands to generate anhydromuropeptides—the exact substrates transported by AmpG. This prediction strongly supports a 'substrate channeling' hypothesis, in which the PPD physically tethers Slt to the membrane surface. This coupling would ensure that muropeptides generated by Slt are immediately delivered to the AmpG1 import channel before they can diffuse away and become diluted within the periplasmic space, thereby maximizing recycling efficiency.

**Table 1:** Candidate periplasmic PG turnover and recycling proteins screened for interaction with AmpG1

Protein	Function	ipTM	pTM
Slt	Soluble lytic transglycosylase	0.55	0.65
PBP7/8 (PBPG)	D,D-endopeptidase	0.29	0.59
RlpA	Septal ring lytic transglycosylase RlpA family	0.26	0.64
L,D-transpeptidase	L,D-transpeptidase	0.26	0.64
LT	Lytic transglycosylase	0.25	0.58
MltE	Soluble lytic transglycosylase domain containing protein	0.25	0.61
PBP1A (*ponA*)	Glycosyltransferase, transpeptidase	0.22	0.45
MrdB (*rodA*)	Glycosyltransferase	0.22	0.56
PBP3 (*ftsI*)	D,D-transpeptidase	0.21	0.48
MltD	Membrane-bound lytic murein transglycosylase D	0.2	0.41
LT2	Lytic transglycosylase	0.19	0.54
PBP1B (*mrcB*)	Glycosyltransferase, transpeptidase	0.19	0.45
PBP6B (*dacD*)	D,D-carboxypeptidase	0.19	0.53
PBP5 (*dacC*)	Serine-type D,D-carboxypeptidase	0.18	0.55
PBP2 (*mrdA*)	D,D-transpeptidase	0.17	0.46
YkuD	L,D-transpeptidase	0.17	0.54
Endopeptidase	Endopeptidase La	0.16	0.45
MltG	Endolytic murein transglycosylase (LT)	0.14	0.6
MltB	Membrane-bound lytic murein transglycosylase	0.14	0.57
MtgA	Glycosyltransferase	0.14	0.61

Candidate proteins were selected from *A*. *baumannii* ATCC 17978 based on predicted periplasmic localization and functional annotation in PG synthesis, turnover, and recycling. Each candidate was modeled with AmpG1 using AlphaFold 3 multimer prediction, and the best ranked model for each pair is summarized. The table reports protein functions, protein annotations, and model confidence metrics including ipTM and pTM scores, which reflect the predicted reliability of the interprotein interface.

To experimentally validate the physical interaction predicted by AlphaFold 3, binding affinity was determined by microscale thermophoresis (MST) using proteins which were overexpressed and then purified. The purified fractions are shown by SDS-PAGE, with His6-Slt lacking the signal peptide and His6-PPD (Fig. S7, *A* and *B*). MST revealed concentration-dependent binding between the PPD and Slt. Specifically, when a fixed concentration of fluorescently labeled PPD was titrated with increasing concentrations of unlabeled Slt, progressive shifts in the thermophoretic traces and a sigmoidal binding curve in the normalized fluorescence were observed, yielding a K_d_ of 5.98 ± 1.74 nM (Fig. 3, *B* and *C*). Isothermal titration calorimetry (ITC) was additionally performed to independently assess this interaction, but the binding curve did not reach saturation under the tested conditions, and the affinity estimate derived from the fit was substantially weaker than that determined by MST, suggesting that the protein system was not optimally amenable to ITC under the tested conditions (Fig. S8). Nevertheless, the thermogram detected heat exchange consistent with an endothermic binding event, providing partial thermodynamic support for the interaction. This coupled mechanism, where a transporter's extracellular domain directly recruits the enzyme that generates its substrate, is analogous to other MFS transporters, such as mammalian PepT1 and PepT2, which also possess extracellular Ig-like domains (31, 32). Biophysical studies revealed that those domains interact with trypsin, the protease that generates their peptide substrates (31).

This clear, dose-dependent sigmoidal curve is experimental proof of a specific and direct *in vitro* interaction between the two proteins. Because *in vitro* assays established direct binding between the AmpG1 PPD and Slt, complex formation in cells was next examined by *in vivo* crosslinking followed by immunoprecipitation. As a preliminary step, anti-FLAG Western blotting of the AmpG1_3×FLAG_ strain was performed to assess protein levels, showing a 1.21-fold increase in AmpG1_3×FLAG_ during stationary phase relative to exponential phase (Fig. 3*D*). This temporal trend, showing a slight upregulation in stationary phase, correlates with the timing of the morphological defects observed in Δ*ampG1* mutant cells (Fig. 1*B*), suggesting that the demand for AmpG1-mediated recycling may be highest during this growth phase. AmpG1_3×FLAG_ migrated at an apparent ∼70 kDa, which is lower than its predicted mass (82.4 kDa), reflecting faster SDS-PAGE mobility driven by strong SDS binding to hydrophobic membrane proteins and producing a downward shift in apparent molecular mass (33). To confirm the *in vivo* complex, intact cells were treated with the chemical crosslinker disuccinimidyl suberate (DSS). Following mild detergent solubilization and anti-FLAG immunoprecipitation, additional high-molecular-mass species above 70 kDa were observed that were not detected without crosslinking (Fig. 3*E*). Notably, proteomic analysis of these immunoprecipitation samples detected Slt, providing evidence that Slt is physically associated with the AmpG1 complex *in vivo* (Table S1). This physical association was further validated using a bacterial adenylate cyclase-based two-hybrid assay. To identify the optimal configuration for interaction, AmpG1 PPD was cloned into the T25-fragment vectors (pKNT25 and pKT25), while Slt was cloned into the T18-fragment vectors (pUT18 and pUT18C). Among the tested fusion combinations, a positive interaction was specifically observed in cells cotransformed with pKT25::*ampG1*^*PPD*^ and pUT18::*slt* (Fig. 3*F*). This result reinforces the direct interaction observed *in vitro* and indicates that the AmpG1 PPD and Slt are positioned to interact functionally within the cellular environment. To exclude nonspecific association with PG-related membrane proteins, AmpG1^PPD^ was paired with membrane-associated proteins of *A. baumannii* involved in PG synthesis (PBP1B and PBP3) or remodeling (PBP5) in the two-hybrid assay. None of these combinations produced a positive signal, further supporting the specific interaction between AmpG1^PPD^ and Slt (Fig. S9). Collectively, these findings provide a direct structural basis for the hypothesized substrate channeling mechanism, which is anticipated to be critical for the overall efficiency of the PG recycling pathway.

### Functional coupling between Slt and AmpG1 in PG recycling

**Figure 4:**
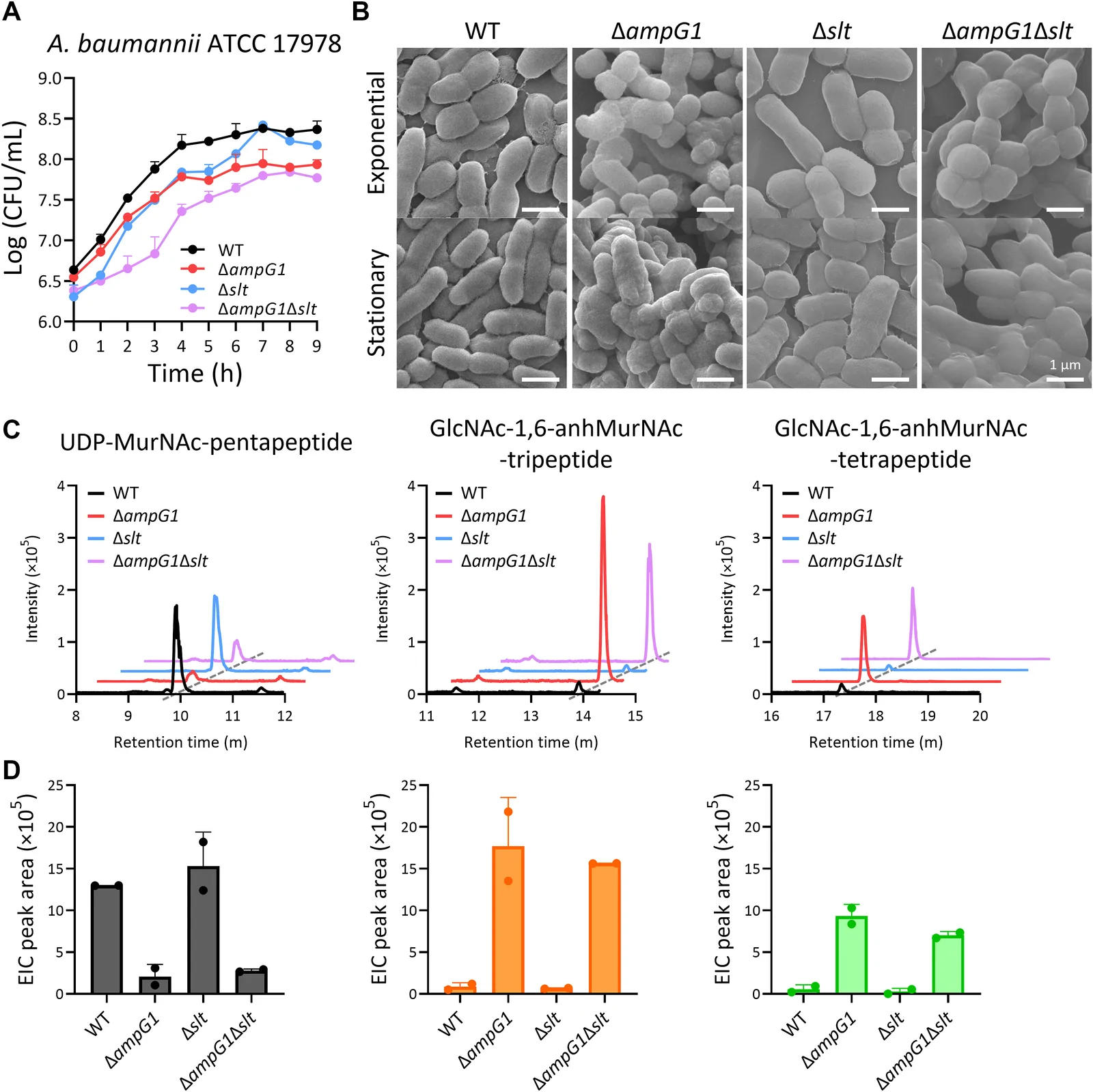
**Indication of AmpG1-Slt coupling for PG recycling in *A. baumannii* by LC-MS/MS analysis.***A*, growth curves of WT, Δ*ampG1*, Δ*slt*, and Δ*ampG1*Δ*slt* in *A. baumannii* ATCC 17978. Data show mean ± s.d. based on n = 3 biological replicates. At time points with no visible bars, the s.d. is smaller than the symbol. *B*, SEM analysis of WT, Δ*ampG1*, Δ*slt*, and Δ*ampG1*Δ*slt* acquired in exponential and stationary phases. Scale bars, 1 μm. *C*, extracted ion chromatograms for UDP-M5, M3N, and M4N in the indicated strains corresponding to (*D*). *D*, targeted LC-MS/MS of cell lysates from stationary phase WT, Δ*ampG1*, Δ*slt*, and Δ*ampG1*Δ*slt*. Bars show amounts of UDP-M5, M3N, and M4N. M3N, GlcNAc-1,6-anhMurNAc-tripeptide; M4N, GlcNAc-1,6-anhMurNAc-tetrapeptide; PG, peptidoglycan; Slt, soluble lytic transglycosylase; UDP-M5, UDP-MurNAc-pentapeptide.

To test the predicted coupling between Slt and AmpG-dependent muropeptide import, Δ*slt* mutant cells and Δ*ampG1*Δ*slt* mutant cells were constructed and compared with Δ*ampG1* mutant cells and WT (Fig. S1). The phenotypes of the single deletion mutants sharply differed, suggesting a principal role for AmpG1 in maintaining cellular integrity. During the exponential phase, Δ*ampG1* and Δ*slt* mutant cells exhibited similar growth rates. However, in the stationary phase, Δ*slt* mutant cells recovered WT-level growth, whereas Δ*ampG1* mutant cells continued to show a significant growth reduction (Fig. 4*A*). Similarly, SEM analysis indicated a WT-like morphology for Δ*slt* mutant cells, in marked contrast to the abnormal cell shape displayed by Δ*ampG1* mutant cells, which became more pronounced in the stationary phase (Fig. 4*B*). The most severe growth phenotype was observed in the Δ*ampG1*Δ*slt* double mutant (Fig. 4*A*), which exhibited a synthetic effect of the dual loss, remaining below both single deletion strains and WT across all growth comparisons. Loss of AmpG1 removes the principal route for muropeptide import, whereas LT redundancy in the Δ*slt* background, including MltABDEFG, likely provides partial compensation (14). Consequently, the double deletion eliminates both import and a major glycan-processing step, restricting PG remodeling and the recycled-precursor supply, thereby producing a stronger growth defect, while residual LT activities and envelope-stress responses may also contribute. The contribution of the AmpG1–Slt complex to PG biosynthesis and recycling was quantified by analyzing cell lysates from four strains (WT, Δ*ampG1*, Δ*slt*, and Δ*ampG1*Δ*slt*) *via* LC-MS/MS (Fig. 4, *C* and *D*; Fig. S10). This analysis targeted the PG precursor UDP-M5 and periplasmic turnover products GlcNAc-1,6-anhMurNAc-tripeptide (M3N) and GlcNAc-1,6-anhMurNAc-tetrapeptide (M4N) to measure precursor and anhydromuropeptide accumulation. The cytosolic precursor UDP-M5 remained near the WT level at 1.18-fold in Δ*slt* mutant cells, indicating that Slt is not essential for precursor generation. Conversely, Δ*ampG1* mutant cells showed a drastic depletion of UDP-M5, dropping to 16% of WT. This confirms that loss of AmpG1 severely depletes the recycled input and compromises the entire cytosolic precursor pool. The strongest accumulation was observed for M3N, which reached 20.65-fold of WT in Δ*ampG1* mutant cells, while the Δ*ampG1*Δ*slt* mutant showed an 11.6% reduction compared to Δ*ampG1* but remained 18.25-fold of WT. A similar trend was observed for M4N, where the double mutant showed a smaller reduction to 75.2% of the Δ*ampG1* level and remained 12.04-fold of WT. These values indicate that the loss of AmpG1 is associated with reduced UDP-M5 levels and marked accumulation of anhydromuropeptides. The lower M3N and M4N levels in the double mutant relative to Δ*ampG1* suggest that Slt loss partially reduces anhydromuropeptide generation, although substantial accumulation remains.

**Figure 5:**
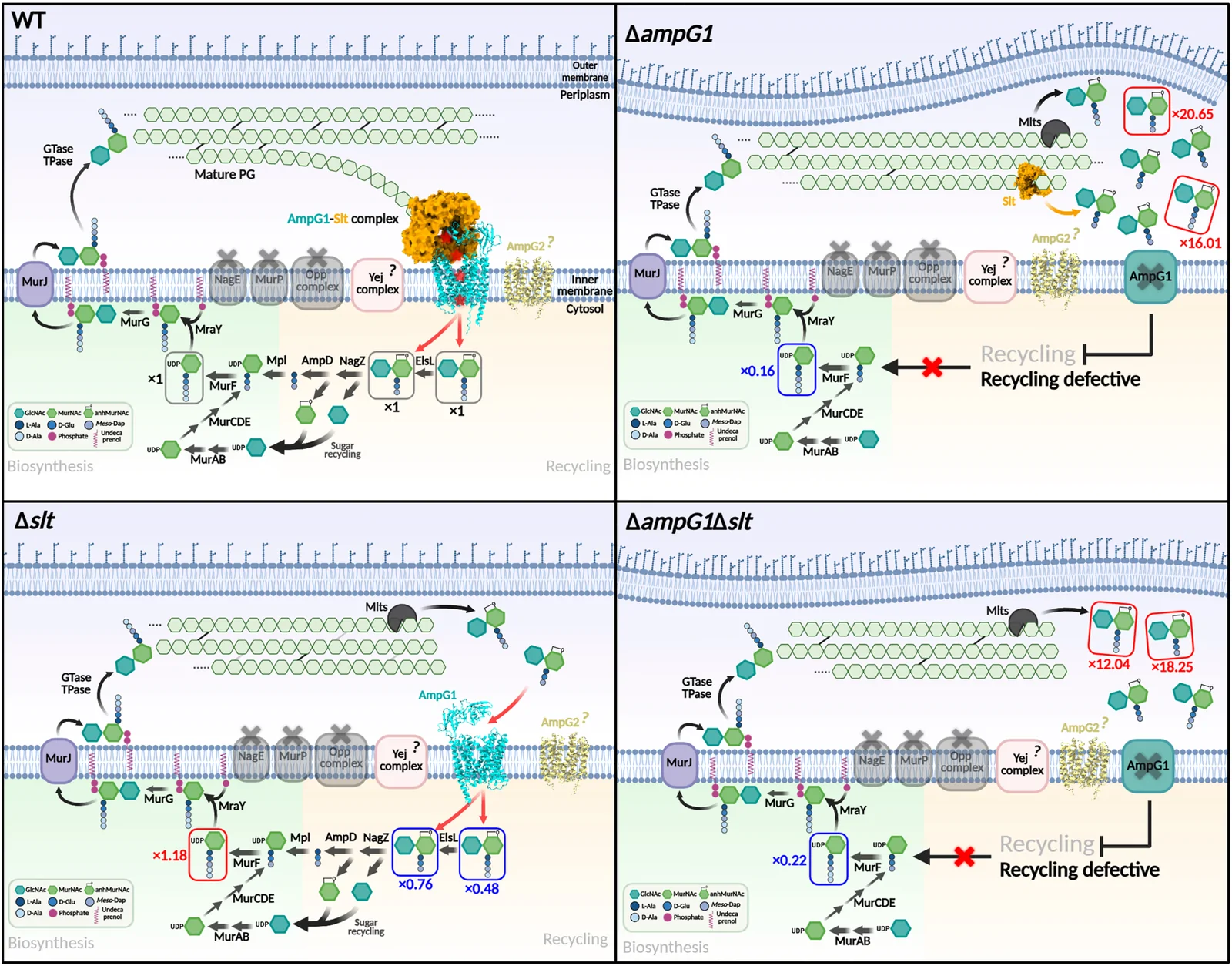
**Schematic scenarios of PG recycling derived from LC-MS/MS composition changes.** Panels depict WT and the Δ*ampG1*, Δ*slt*, and Δ*ampG1*Δ*slt* mutant strains. *Blue text* denotes fold-change decreases relative to WT, and *red text denotes* fold-change increases relative to WT. Numerical labels indicate relative changes at the annotated steps inferred from LC-MS/MS analysis. PG, peptidoglycan.

A schematic scenario derived from LC-MS/MS composition changes summarizes PG recycling in WT and in the Δ*ampG1*, Δ*slt*, and Δ*ampG1*Δ*slt* mutant strains (Fig. 5). In WT, AmpG1 binds Slt at the inner membrane through its PPD, channels newly generated anhydromuropeptides into the importer for transport, and allows ElsL, NagZ, AmpD, and Mpl to regenerate cytosolic precursors that sustain biosynthesis (19, 22). A diffusion-mediated route may operate in parallel, and our data indicate that coupling between AmpG1 and Slt constitutes the predominant route. The functional loss of AmpG1 disables muropeptide import while Slt continues to generate anhydromuropeptides, a conflict that causes the accumulation of GlcNAc-1,6-anhMurNAc-peptides and related fragments within the periplasm, reduces the inflow of recycled precursors, and produces a functional defect in recycling. In the Δ*slt* mutant cells, AmpG1 retains import competence while Slt-dependent generation of anhydromuropeptides declines, reducing the supply of recycled material. Nevertheless, the UDP-M5 pool is maintained, consistent with continued precursor production through *de novo* biosynthesis. In the Δ*ampG1*Δ*slt* mutant cells, both import and Slt-dependent generation are impaired, and recycling remains defective, as indicated by the persistently reduced level of UDP-M5. However, M3N and M4N remain strongly elevated, indicating that Slt is a major, but not exclusive, source of anhydromuropeptides and that additional lytic transglycosylases likely contribute to their generation in the absence of Slt. Together, these patterns support a coupled delivery model in which Slt locally generates anhydromuropeptides and AmpG1 captures and imports them, coordinating PG recycling.

## Discussion

In gram-negative bacteria, PG recycling is central to envelope stability and antibiotic tolerance, yet whether periplasmic turnover and cytosolic import are physically coupled remains uncertain (34, 35, 36). In model organisms like *E. coli*, muropeptides released by LTs are thought to diffuse through the periplasm before being captured by the permease AmpG (15). This diffusional separation is a key factor in the balance between PG synthesis and stress signaling. This model is not the only one, as *E. coli* also employs a coupled uptake route for oligopeptides *via* the OppBCDF ABC transporter, which relies on the soluble periplasmic protein MppA to capture and deliver substrates (37, 38). The *A. baumannii* system identified here represents a third, distinct strategy, achieving a coupled import like MppA-Opp but through a novel architecture where the adapter domain is integrated directly into the MFS transporter. To determine the physiological consequences of disrupting this Slt-AmpG1 route, β-lactam MICs were analyzed as a functional measure of AmpG1-dependent import (Fig. S11*A*). The standard mechanism for β-lactam resistance in *P. aeruginosa* is AmpR-mediated β-lactamase induction (20). In this model, β-lactam exposure causes AmpG to import GlcNAc-1,6-anhydroMurNAc-pentapeptide, which NagZ then converts into the species that activates AmpR to drive *ampC* expression. Because *A. baumannii* lacks *ampR*, β-lactamase activity assays were performed to rule out this alternative mechanism; the results detected no differences between WT and Δ*ampG1* mutant cells (Fig. S11*B*), thereby confirming the hypersusceptibility is due to defective PG recycling (39). β-lactamase activity assays under ampicillin, amoxicillin, and cefotaxime, as well as in the untreated condition, detected no differences between WT and Δ*ampG1* mutant cells (Fig. S11*B*). Ethidium bromide and 1-N-phenylnaphthylamine (NPN) uptake measurements demonstrated lower overall membrane permeability in Δ*ampG1* mutant cells than in WT, arguing against increased permeability or efflux-driven uptake as the basis for the MIC decrease (Fig. S11*C*) (40, 41). The most consistent interpretation is that reduced MICs arise from a reduced supply of PG precursors and the periplasmic accumulation of turnover products due to impaired AmpG1-dependent muropeptide import. A similar hypersensitivity phenotype was reported in *Caulobacter crescentus* following *ampG* deletion, supporting a conserved link between defective recycling and weakened β-lactam tolerance across phylogenetically distant gram-negative species (42).

Comparative genomic analysis reveals that *A. baumannii* employs a complex PG recycling strategy, characterized by the retention and expansion of multiple, distinct transporter families. Whereas many enterobacteria typically encode a single *ampG* gene (21), *A. baumannii* carries three homologs and also encodes the ABC transporter YejABEF (14), which is analogous to the YejBEF–YepA complex in *Agrobacterium tumefaciens* that transports anhydromuropeptides generated during PG turnover (43). This genetic redundancy may compensate for the notable absence of a defined periplasmic N-acetylmuramyl-L-alanine amidase in *A. baumannii* (44). Without this enzyme, removal of the peptide stem may be infrequent, meaning disaccharide–peptide species would need to be imported intact. These features likely increase reliance on dedicated import routes. Consideration was then given to how different *ampG* homologs contribute to this route. The data distinguish AmpG1, a 14-TM permease with a periplasmic insertion, from AmpG2, a shorter homolog that lacks this insertion and remains largely silent under the tested growth conditions (Fig. 1, *A*–*D*). The Δ*ampG1*Δ*ampG2* mutant cells show a composite phenotype that does not simply recapitulate either single deletion. Significantly, the double mutant grew to near-WT levels (Fig. S3*A*) and displayed β-lactam MICs 2- to 16-fold higher than WT (Fig. S3*B*). The Δ*ampG1*Δ*ampG3* mutant cells showed β-lactam MICs higher than those of the Δ*ampG1* mutant and similar to WT, whereas Δ*ampG3* and Δ*ampG2*Δ*ampG3* mutant cells had MICs equal to WT (Fig. S3*B*). The Δ*ampG1*Δ*ampG3* mutant was also less sensitive to lysozyme and fosfomycin than the Δ*ampG1* mutant, though it remained more sensitive than WT (Fig. S3, *C* and *D*). For fosfomycin, the MIC of the Δ*ampG1*Δ*ampG3* mutant was 256 μg/ml or higher, compared with 32 μg/ml for the Δ*ampG1* mutant (Fig. S3*D*). However, this overall trend toward reduced antibiotic sensitivity was contrasted by a marked hypersensitivity to EDTA under the same conditions (Fig. S3*E*). Considered together, these observations indicate outer membrane remodeling that modifies barrier properties and antibiotic entry modes, although the underlying mechanism remains to be defined. The patterns further suggest that the simultaneous loss of both transporters prompts envelope adjustments that are distinct from either single deletion.

This entire gram-negative recycling system contrasts with PG metabolism in gram-positive bacteria, which lack an outer membrane and a defined periplasmic compartment (1). AmpG-type transporters are largely absent in gram-positive species, so periplasmic recycling as defined for gram-negative cells does not occur (2). Instead, gram-positive species rely on a carbohydrate-centered salvage strategy. Cell-wall hydrolases release PG fragments to the medium, from which specific sugars like MurNAc are subsequently recovered for transport (45). A key example is the MurP transporter, which imports MurNAc that is then immediately processed by the cytoplasmic kinase MurQ for entry into sugar recycling pathways (45). Consistent with this sugar-focused approach, peptide moiety is generally not imported as an intact muropeptide. Gram-negative bacteria utilize the Mpl to re-attach imported peptides, but this enzyme is absent in most gram-positive species (4, 46). Together, these distinctions indicate that while PG remodeling is widespread, its spatial organization differs fundamentally. Gram-negative species integrate periplasmic hydrolysis with inner-membrane import, whereas gram-positive species rely on extracellular release and cytoplasmic sugar salvage.

A key question is whether the addition of large functional domains to AmpG is a widespread evolutionary strategy or a rare, lineage-specific adaptation like the AmpG1^PPD^. To investigate whether similar extended-length AmpG homologs exist in other species, an HMM profile was constructed based on conserved AmpG sequences from the Swiss-Prot database. This profile was then used to search the UniProtKB (Swiss-Prot and TrEMBL) databases, yielding 18,392 AmpG-like sequences, of which 334 were longer than 650 aa. Structural predictions using AlphaFold 3 indicated that these homologs also possess extra domains. However, these were typically located at the N terminus, unlike the internal domain of AmpG1. Furthermore, phylogenetic analysis revealed that these extended-length AmpG proteins are not broadly conserved, suggesting they arose from multiple, independent evolutionary events (Fig. S12). This finding suggests that while the MFS transporter core is ancient, the addition of large functional domains such as the PPD in AmpG1 is a more recent, lineage-specific adaptation rather than a common evolutionary strategy. The complete absence of large multicomponent transporters like the Opp ABC family defines a recycling system in *A. baumannii*, a simplified uptake strategy centered on AmpG that could be interpreted as an evolutionary trade-off (14). Maintaining large, multiprotein complexes like ABC transporters incurs a substantial bioenergetic cost, both in terms of gene replication and protein translation (47, 48). It is plausible that *A. baumannii* evolved a more economical system by forgoing these "expensive" transporters and instead investing in a modified MFS transporter (AmpG1) that was "upgraded" with the PPD to achieve the high-efficiency import required for its load-bearing role. The evolutionary pressure to develop such a specialized capture mechanism is underscored by the recent discovery that muropeptides are a valuable public good for which bacteria compete in microbial ecosystems. Some bacteria, like '*Candidatus* Marinimicrobia' (Marine Group A), have even lost *de novo* PG synthesis entirely and survive by scavenging muropeptides released by other bacteria (49). This extreme dependency highlights an evolutionary landscape where PG fragments are a shared resource. In this context, the Slt–AmpG1 complex is not just an internal recycling machine but represents a sophisticated competitive adaptation to ensure *A. baumannii* can efficiently capture and privatize this public resource before it is lost to diffusion or consumed by competitors. The identification of the PPD-Slt complex reveals a novel substrate-channeling mechanism that is critical for *A. baumannii*'s cell wall homeostasis. Because this protein–protein interaction is both essential for the primary recycling pathway and highly specific—mediated by a genus-specific Ig-like domain—it represents a promising new class of drug target.

Mechanistic insight into how AmpG1 and Slt associate remains incomplete, as the molecular determinants governing their interaction have yet to be defined. Future studies should employ structure-guided point mutations targeting the predicted interaction surfaces of the AmpG1 PPD and Slt and evaluate their effects on complex formation and associated cellular phenotypes. In parallel, quantitative affinity measurements for relevant muropeptide substrates (commercially not available) will be important to determine whether coupling alters substrate binding and handling in a way that can explain pathway-level efficiency. Beyond Slt, *in vivo* crosslinking followed by anti-FLAG immunoprecipitation and proteomic analysis identified multiple proteins associated with AmpG1 in cells. Notably, several divisome-related proteins, including FtsZ, ZapE, and FtsI, were among the candidate interactors, raising the possibility that the AmpG1–Slt module is spatially positioned near the division machinery or engages divisome-associated factors (Fig. 3*E*; Table S1). Such an arrangement could facilitate coordination of localized PG turnover and import with septal PG synthesis during cell division. However, because crosslinking-based proteomics can capture both direct interactors and proteins in close spatial proximity, the specificity and functional relevance of these associations remain to be established. Elucidating these molecular networks will provide a more comprehensive understanding of how *A. baumannii* coordinates cell wall recycling with growth and division. Building on this mechanistic foundation, the unique and genus-specific nature of the AmpG1–Slt complex presents an attractive opportunity for therapeutic intervention.

## Experimental procedures

### Bacterial strains and growth conditions

*A. baumannii* ATCC 17978 obtained from the ATCC was used as the reference strain and was maintained in the laboratory. In this study, the three *ampG* homologs in *A. baumannii* ATCC 17978 are designated *ampG1* (locus tag E5A70_06500), *ampG2* (E5A70_05795), and *ampG3* (E5A70_04775). All the strains were grown in LB broth at 37 °C with aeration by shaking at 180 rpm, unless otherwise noted. Growth was monitored by plate counts and expressed as log10 (CFU/ml). LB agar was supplemented with antibiotics (kanamycin at 50 μg/ml and apramycin at 100 μg/ml). All knock-out strains were constructed using CRISPR-Cas9. The *ampG1*-complemented strain was generated by cloning its native promoter, the *ampG1* coding sequence, and a C-terminal 3×FLAG tag into pBBR1MCS-2. The plasmid construct was verified by PCR and sequencing using primers listed in Table S2, after which electrocompetent Δ*ampG1* cells were prepared and transformed by electroporation. Transformants were selected on LB agar containing kanamycin (50 μg/ml), and the insert was re-confirmed by colony PCR.

### Construction of knockout strains using CRISPR-Cas9

CRISPR-Cas9 gene deletions (Δ*ampG1*, Δ*ampG2*, Δ*ampG3*, Δ*ampG1*Δ*ampG2,* Δ*ampG1*Δ*ampG3,* Δ*ampG2*Δ*ampG3,* Δ*slt,* and Δ*ampG1*Δ*slt*) were generated in *A. baumannii* ATCC 17978 using pCasAb-apr (Addgene 121998) and pSGAb-km (Addgene 121999) as described (50, 51, 52). Briefly, 20 bp spacer oligonucleotides and 80 nt donor oligonucleotides were designed for each locus (Table S2). Phosphorylated spacers were inserted into pSGAb-km by Golden Gate assembly, transformed into *E. coli* DH5α, selected on LB agar with kanamycin (50 μg/ml), and verified by PCR and M13R sequencing. For editing, cells harboring pCasAb-apr were induced with IPTG to express RecAb and Cas9, made electrocompetent, and co-electroporated with the spacer-bearing pSGAb-km and the single-stranded donor DNA. Transformants were selected on LB agar with apramycin (100 μg/ml) and kanamycin (50 μg/ml) at 37 °C overnight. Correct deletions were confirmed by diagnostic PCR and sequencing, and plasmids were cured by growth on 5% sucrose followed by confirmation of antibiotic sensitivity.

### Antibiotic susceptibility tests

MICs were determined by broth microdilution in LB with *A*_600_ readout. Ampicillin, amoxicillin, cefotaxime, cephalexin, meropenem, and fosfomycin were tested in 2-fold serial dilutions; for all agents except fosfomycin, the range was 0.125 to 256 μg/ml, whereas fosfomycin dilutions started at 512 μg/ml. Following inoculation, plates were incubated at 37 °C for 24 h under static conditions, and *A*_600_ was measured using a Spark microplate reader (Tecan, Switzerland). Experiments were performed with ≥3 biological replicates.

### Prediction of protein structure and topology analysis

AlphaFold 3 was used to predict the structures of AmpG1 and AmpG2 and the complex between the AmpG1 and Slt (53). Predictions were run with default settings, and pTM and ipTM scores were recorded for quality assessment. The resulting models were inspected, colored, and rendered in ChimeraX (54). Membrane placement of the AlphaFold 3 models was performed with PPM 2.0 (55). PPM 2.0 outputs were opened in ChimeraX for editing and visualization. Sequence-based topology was obtained with Protter using default parameters from the primary sequence (56). Across methods, the inserted domain of AmpG1 was placed on the periplasmic side of the inner membrane, supporting its assignment as a PPD.

### PG isolation and LC-QTOF-MS/MS analysis

Cells were cultured to stationary phase (500 ml per prep; Δ*ampG1* in 2-L flasks for adequate aeration), harvested by cold centrifugation, and washed twice with ice-cold PBS. Preparation of sacculi followed a published protocol with minor modifications (57). Pellets were resuspended in 0.25% (w/v) SDS prepared in 0.1 M Tris-HCl pH 7.6 and boiled at 100 °C for 20 min. All samples and enzymes were incubated using Thermomixer model C (Eppendorf) with shaking at 500 rpm. Insoluble sacculi were collected by centrifugation and washed six times with ice-cold deionized water to remove SDS, then briefly probe-sonicated (2 s pulse/4 s rest, 40% amplitude, three cycles). Samples were treated with DNase (Sigma-Aldrich; 15 μg/ml) and RNase (Sigma-Aldrich; 60 μg/ml) at 37 °C for 1 h, followed by trypsin (Sigma-Aldrich; 50 μg/ml) and proteinase K (NEB; 100 μg/ml) at 37 °C for 1 h. Enzymes were heat-inactivated at 100 °C for 3 min, and samples were washed once with water. Sacculi were resuspended in 1 M HCl and incubated at 37 °C for 4 h, then washed five times with water to neutrality. Purified PG was digested with lysozyme (2 mg/ml) at 37 °C for 16 h with shaking at 500 rpm, heated at 100 °C for 3 min to stop the reaction, clarified by centrifugation, and the supernatant was filtered through a 0.45 μm membrane. Soluble muropeptides were either stored at 4 °C for up to 1 week or subjected immediately to LC-QTOF-MS/MS analysis.

Purified PG samples were analyzed by LC-QTOF-MS/MS analysis using an Agilent 1290 Infinity II coupled to an AB Sciex Q-TOF 5600+ and a Waters ACQUITY BEH C18 column (1.7 μm, 2.1 × 150 mm) held at 50 °C with a flow of 0.30 ml/min. Mobile phases were 0.1% formic acid in water (A) and 0.1% formic acid in acetonitrile (B). The gradient was as follows: 0 to 3 min, 5% B; 6.0 min, 6.8% B; 7.5 min, 9% B; 9.0 min, 14% B; 11.0 to 12.0 min, 20% B; 12.1 to 13.5 min, 90% B; 13.6 to 18.0 min, 2% B; 18.1 to 20.0 min, 5% B (re-equilibration). The MS was operated with curtain gas 25, GS1 50, GS2 50, ion spray voltage 5500 V, source temperature 500 °C, dynamic collision energy, declustering potential 80 V, TOF scan m/z 50 to 2000, and time bins to sum = 4. The peaks (m/z) corresponding to purified PG were identified based on previous studies (22, 58, 59).

### HADA labeling and confocal microscopy

Overnight seed cultures were used to start exponential-phase cultures at 37 °C with aeration. To equalize cell numbers, WT was grown for 3 h and Δ*ampG1* for 4 h before labeling. Ten minutes prior to harvest, sterile 5-mL conical tubes were prewarmed at 37 °C with 10 μl of HADA stock (1 mM). Ice-cold, pH-adjusted PBS and 100% ethanol were prepared on ice.

Cells were pelleted by centrifugation, 3.5 ml of supernatant was removed, the pellet was resuspended, and 990 μl of the suspension was transferred into the prewarmed tube containing HADA. The final HADA concentration during labeling was 10 μM in a 1 ml reaction. Labeling was carried out at 37 °C with shaking for 2 min 28 s for WT or 5 min 07 s for Δ*ampG1*, which corresponds to about 8% of each strain’s doubling time (WT ≈ 31 min; Δ*ampG1* ≈ 64 min). Fixation was performed by adding 2.3 ml of ice-cold 100% ethanol to reach 70% (v/v) final ethanol, followed by incubation on ice for 10 min. Cells were pelleted, transferred to microcentrifuge tubes, and washed twice with cold PBS. For imaging, cells were mounted on PBS-agarose pads and imaged on a confocal laser scanning microscope (LSM 700, Carl Zeiss). HADA signal was acquired with 405-nm excitation and emission collected near 490 nm using identical acquisition settings across samples. Image processing and fluorescence-intensity measurements were performed in ZEN 3.3 (blue edition). Procedures were adapted from previously published methods (29, 57, 60).

### Bocillin fluorescence binding assay

Cells were grown to stationary phase, and cell densities were equalized by *A*_600_ prior to labeling. Cultures were harvested at 4 °C (max speed, 10 min), washed with ice-cold PBS (10 ml, max speed, 5 min), resuspended in 1 ml PBS, transferred to microcentrifuge tubes, and washed once more (max speed, 2 min). For labeling, pellets were resuspended in 500 μl PBS and mixed with Bocillin-FL (BOCILLIN FL Penicillin, Invitrogen; 1 mg/ml stock thawed at room temperature; 5 μl per 500 μl suspension). Samples were protected from light and incubated for 30 min at room temperature. Cells were pelleted and washed twice with cold PBS (1 ml each, max speed, 2 min). Pellets were resuspended in 800 μl PBS, lysozyme (10 mg/ml stock) was added to 200 μl (final volume 1 ml), and samples were incubated at 37 °C for 30 min in the dark. Cells were disrupted by probe sonication (2 min total per round, 2 s pulse/4 s rest, 40% amplitude) for three cycles on ice. Lysates were cleared by centrifugation (21,000 g, 4 °C, 30 min), and supernatants were discarded to retain the membrane-enriched pellet. Pellets were resuspended in 50 μl PBS, total protein was determined by Bradford assay, and samples were adjusted to 0.8 to 0.9 mg/ml. For SDS–PAGE, 5×SDS sample buffer was added to a 1 × final concentration, and samples were heated at 95 °C for 5 to 10 min and cooled on ice. Bocillin-labeled PBPs were resolved on 10% SDS–PAGE gels with 25 μl per lane (100 V, ∼150 min). In-gel fluorescence was acquired on a Typhoon FLA 9500 (GE Healthcare) using a FITC-compatible channel (excitation ∼488 nm, emission ∼530 nm). Results are reported from replicate experiments performed with matched total protein loading, adapting previously described procedures (57, 61).

### Overexpression and purification of His-tagged protein

The *slt* coding region lacking the N-terminal signal peptide (PCR amplicon 1872 bp) and the *ampG1* PPD (606 bp) were amplified with Phusion Plus PCR Master Mixes (Thermo Fisher Scientific). Both inserts were cloned into pET-28b(+) to place an N-terminal His6 tag on each protein. Constructs were sequence verified and transformed into *E. coli* BL21(DE3). For overexpression, single colonies were inoculated into LB with kanamycin (50 μg/ml) and grown at 37 °C with shaking (180 rpm) to mid-log phase. Protein production was induced and carried out at 16 °C for 20 h under the following conditions: SltΔ*signal-peptide*, IPTG 0.4 mM; AmpG1 PPD, IPTG 1 mM. Cell pellets were washed once with a cold buffer and resuspended in a lysis buffer (20 mM Tris-HCl pH 7.6, 200 mM NaCl; 0.2-μm filtered). Cells were disrupted by probe sonication on ice and clarified by centrifugation (21,000*g*, 4 °C, 30 min). The supernatants were applied to Ni-NTA resin using the HisPur Ni-NTA Purification Kit (Thermo Fisher Scientific) according to the manufacturer’s instructions. For Slt^Δ*SP*^, the column was washed with imidazole at 20 mM and 75 mM in lysis buffer; for AmpG1 PPD, washes used 20 mM and 60 mM imidazole. Proteins were eluted with 250 to 300 mM imidazole, analyzed by SDS-PAGE, and stored as described. Procedures were adapted from Park *et al*. (62).

### Microscale thermophoresis

MST was performed following established protocols with minor modifications (63, 64). His6-Slt lacking the signal peptide and His6-PPD were purified. Imidazole was removed, and samples were buffer exchanged into PBS using Amicon Ultra-4 10K centrifugal filters (Millipore). For the Slt titration series, the His6 tag on Slt was cleaved with thrombin at 10 U per mg protein at 4 °C for 16 h without shaking. Cleavage was verified by a 2.2 kDa band shift on a 7.5% SDS-PAGE gel, then the sample was washed again with Amicon filters.

PPD was labeled with the Monolith His-Tag labeling Kit RED-tris-NTA (NanoTemper Technologies). After 30 min incubation at room temperature, the reaction was centrifuged at 15,000*g* for 10 min at 4 °C, and the supernatant was used for assays. A concentration gradient of tag-free Slt was prepared in PBS and mixed with a fixed amount of labeled PPD. Mixtures were incubated at room temperature for 1 h and loaded into Monolith NT.115 capillaries (NanoTemper Technologies). MST was recorded on a Monolith NT.115 instrument at 25 °C with MST power 50% and excitation power 50%. Data were analyzed in MO.Affinity Analysis from NanoTemper Technologies.

### Isothermal titration calorimetry

ITC was carried out on a MicroCal PEAQ-ITC system (Malvern Panalytical). His6-Slt lacking the signal peptide and His6-AmpG1^PPD^ were overexpressed and purified as described above, and both proteins were buffer-exchanged into PBS (pH 7.4) to minimize buffer-mismatch heats. AmpG1^PPD^ was loaded into the syringe and titrated into Slt held in the sample cell at 25 °C. The titration comprised 19 sequential injections, each of 4.0 s duration, spaced 120 s apart, with a stirring speed of 750 rpm, and a reference power of 5.00 μcal/s. The heat of dilution was subtracted before analysis, and data were processed in the MicroCal PEAQ-ITC analysis software.

### Bacterial two-hybrid system

Protein–protein interactions were assessed using the adenylate cyclase-based bacterial two-hybrid system. Coding sequences of the proteins or domains of interest were cloned in-frame into the T25 and T18 fusion vectors pKT25 or pKNT25 and pUT18 or pUT18C, respectively. Fusion junctions were designed to preserve reading frame and to minimize disruption of predicted structural elements. All constructs were verified by PCR and sequencing. Pairs of T25 and T18 plasmids were cotransformed into *E. coli* BTH101, and transformants were selected on LB agar containing ampicillin at 100 μg/ml and kanamycin at 50 μg/ml at 37 °C. Cotransformant colonies were screened by colony PCR using vector-specific primer sets to verify that each fusion plasmid carried the correct insert. Verified colonies were re-streaked on indicator plates consisting of LB agar supplemented with ampicillin at 100 μg/ml, kanamycin at 50 μg/ml, IPTG at 40 μg/ml, and X-gal at 0.5 mM, followed by incubation at 30 °C for 36 h. Empty vector combinations were included as negative controls, and known interacting pairs supplied with the system were used as positive controls (65). Where relevant, both N-terminal and C-terminal fusion orientations were evaluated to mitigate topology-dependent false negatives, and the fusion combinations used for figure presentation were those providing consistent results across independent replicates.

### Preparation of antibody–bead conjugates

Dynabead protein A 1.5 mg/50 μl (Invitrogen) were washed three times with ice-cold buffer containing 20 mM Hepes pH 7.5 and 150 mM NaCl, then incubated with anti-DDDDK tag antibody (Abcam) on a rotator at 4 °C for 60 min. Antibody-bound beads were crosslinked with DSS (Thermo Scientific) (27.1 mM in DMSO) to a final 2 mM for 30 min at room temperature and quenched with 1 M Tris-HCl pH 7.5 to a final 50 mM for 15 min. Beads were washed and stored in “0% DDM (*N*-dodecyl-β-D-maltoside) buffer” [20 mM Hepes pH 7.5, 300 mM NaCl, 5% glycerol, 100X Protease Inhibitor Cocktail (GenDEPOT)] at 4 °C. Use of glycerol-containing buffers reduces bead adhesion to plastic.

### *In vivo* crosslinking

Δ*ampG1*/pBBR1MCS-2::P*ampG1*-*ampG1*-3 × FLAG were grown in LB, harvested at stationary phase, washed three times with ice-cold PBS, and resuspended in PBS to a total volume of 5 ml in a 15-mL tube. DSS was added to a final 2.5 mM, and the suspension was rotated gently for 30 min at room temperature. Crosslinking was quenched with 1 M Tris-HCl pH 7.5 to a final 50 mM for 15 min with gentle rotation. Cells were washed twice with ice-cold PBS. Procedures were adapted from Castanheira *et al*. (66).

### Cell lysis and membrane solubilization

Cell disruption, membrane isolation, and DDM (Sigma-Aldrich) solubilization were performed essentially as described previously with minor modifications (20). Hepes-based buffers (20 mM, pH 7.5) containing 150 mM NaCl and 5% glycerol, addition of 2 mM MgCl_2_ and DNase I at 20 μg mL^−1^, sonication on ice at 20% amplitude in 1 s/2 s cycles for 4 min, differential centrifugation at 15,000×*g* for 20 min followed by 200,000*g* for 1 h, and membrane solubilization in 1% DDM with 300 mM NaCl for 60 min on ice before clearing by high-speed centrifugation for 30 min at 4 °C.

### Anti-FLAG immunoprecipitation under mild detergent

For binding, 500 μl of the 1% DDM solubilized sample was mixed with 3.5 ml of 0% DDM buffer to obtain 0.1% DDM, and 1 ml of DSS-crosslinked anti-FLAG–bead slurry was added. The mixture was rotated at 4 °C for 4 h. Beads were captured on a magnetic rack, and the complexes were washed sequentially with two detergent-containing buffers: first with 0.1% DDM wash buffer (20 mM Hepes pH 7.5, 300 mM NaCl, 5% glycerol, and protease inhibitor), then with 0.05% DDM wash buffer (20 mM Hepes pH 7.5, 200 mM NaCl, 5% glycerol, and protease inhibitor). Bead-bound proteins were eluted by adding 1 × Laemmli sample buffer prepared fresh from sample buffer (Laemmli's 5X) (ELPIS-BIOTECH) and heating at 70 °C for 10 min. Samples were resolved on 8% SDS-PAGE gels and transferred at 30 V for 150 min. Blots were probed with anti-FLAG (1:5000) followed by HRP-conjugated secondary antibody (1:5000). Chemiluminescent signals were detected and analyzed using an iBright CL1500 Imaging System (Invitrogen). Procedures were adapted from Castanheira *et al*. (66).

### *β-*Lactamase enzymatic activity

β-Lactamase activity was assessed by nitrocefin hydrolysis as described previously (67). Cells were grown aerobically at 37 °C for 7 h, then exposed to the indicated β-lactam at one-fourth MIC for 2 h to induce β-lactamase expression. Cell densities were normalized by *A*_600_, harvested by centrifugation, and washed twice with 0.1 M sodium phosphate buffer (pH 7.0). Pellets were resuspended in the same buffer and lysed by probe sonication on ice (short pulses with cooling). Lysates were clarified by centrifugation (≈18,000*g*, 30 min, 4 °C), and the supernatant was used as the enzyme source. For the chromogenic reaction, 100 μl of lysate was mixed with 75 μl phosphate buffer and nitrocefin (final 0.5 mg/ml, Abcam) in 96-well plates. Absorbance at 486 nm was recorded kinetically at 37 °C on a Spark microplate reader (Tecan) at 5-min intervals for 60 min.

### Membrane permeability assay

EtBr uptake assays were performed on stationary phase cultures as described previously with minor modifications (57). Cells were harvested, washed twice in 50 mM K-phosphate buffer pH 7.0, and normalized. Where indicated, CCCP (100 μM) was added for 5 min to collapse the proton motive force. Ethidium bromide was then added to a final concentration of 5 μg/ml, and fluorescence of the EtBr–nucleic acid complex was recorded at 37 °C in black 96-well plates (Spark microplate reader, Tecan; λ_ex 515 nm, λ_em 590 nm). Signals were background-subtracted and normalized to *A*_600_. Measurements were performed in triplicate.

NPN uptake assays were performed on stationary phase cultures as described previously with minor modifications (57). Cells were harvested, washed twice, and resuspended in 5 mM Hepes pH 7.2, and normalized. Immediately before reading, NPN was added to 20 μM, and fluorescence was measured within 3 min at 37 °C (Spark microplate reader; λ_ex 340 nm, λ_em 405 nm). Data were collected in triplicate and reported as background-subtracted fluorescence normalized to *A*_600_.

### Analysis of PG precursor and muropeptides

Cultures were grown at 37 °C with aeration to stationary phase. Cell lysate preparation was adapted from Dai *et al*. (22). Cell densities were normalized by *A*_600_ across conditions prior to extraction. Pellets were washed twice with sterile 1% NaCl (0.2-μm filtered). Pellets were resuspended in 4 ml deionized water and heated at 100 °C with agitation (1000 rpm) for 50 min using Thermomixer model C (Eppendorf). Suspensions were centrifuged at 15,000 rpm for 20 min at 4 °C, supernatants were collected, passed through 0.2-μm membrane filters, and stored at 4 °C. These clarified cell lysates were analyzed directly to quantify PG precursors and turnover products in the same run. Analyses were performed on an Agilent 1290 Infinity II coupled to an AB Sciex TripleTOF 5600+ with a Waters ACQUITY BEH C18 column (1.7 μm, 2.1 × 150 mm), held at 45 °C and 0.30 ml/min. Mobile phases were 0.1% formic acid in water (A) and 0.1% formic acid in acetonitrile (B). Gradient: 0 to 3 min, 1% B; 30 min, 12% B; 34 min, 20% B; 35 to 37 min, 80% B; 38 to 40 min, 1% B (re-equilibration). The MS was operated in positive ESI with curtain gas 25, GS1 50, GS2 50, ion spray voltage 5500 V, source temperature 500 °C, dynamic collision energy, declustering potential 80 V, TOF scan m/z 200 to 2000, and time bins to sum = 4. Targeted species were evaluated by extracted-ion chromatograms and assigned by accurate mass.

### Phylogenetic analysis of ampG homologs

To construct an HMM profile for AmpG, an initial set of 20 sequences annotated as AmpG was retrieved from Swiss-Prot. These sequences were aligned using MAFFT (--auto), and the resulting alignment was used as input for the hmmbuild program (HMMER suite) (68, 69). This profile was then used to search the Swiss-Prot and TrEMBL databases with hmmsearch, identifying 18,392 AmpG-like sequences. This set was subsequently clustered at 50% sequence identity using CD-HIT to generate 1075 representative AmpG sequences (70). A multiple sequence alignment of these representative sequences was then constructed with MAFFT (L-INS-i) and trimmed using trimAl using a 90% gap threshold (71). The final alignment was used for phylogenetic inference in IQ-TREE 3.0.1 with the LG + F + R10 model, as selected by ModelFinder (72, 73). The resulting tree was annotated and visualized using iTOL v7 (74).

### Statistical analysis

Unless otherwise stated, experiments were performed with at least three biological replicates, and results are presented as the mean ± standard deviation. The LC-MS/MS analyses of PG composition and of cytosolic precursors and turnover products were performed with two independent biological replicates, and individual replicate values are shown alongside the mean. Analyses were performed using GraphPad Prism, version 9.1.0 build 221, and Microsoft Office 365 for Windows.

## Data availability

The datasets used in this study are available in the NCBI sequence archives under the accession number CP039028.2 (*A. baumannii* ATCC 17978 substr. Lab-WT chromosome). The *in vivo* crosslinking proteomics dataset has been deposited in iProX/ProteomeXchange under accession PXD072474 (iProX: IPX0014868001).

## Supporting information

This article contains supporting information.

## Conflict of interest

The authors declare that they have no conflicts of interest with the contents of this article.
