# Supplementary material for: Peptidoglycan permease AmpG1 couples lytic transglycosylase activity to muropeptide import in Acinetobacter baumannii
Source: J Biol Chem. 2026 Jul 24;302(9):113362. doi: 10.1016/j.jbc.2026.113362 (PMC13507758; doi:10.1016/j.jbc.2026.113362)
Supplement: Supplementary file 1 [file mmc1.docx]

**Peptidoglycan permease AmpG1 couples lytic transglycosylase activity to muropeptide import in *Acinetobacter baumannii***

**Taeyeong Kim, Reagan Lee, Yerim Park, Jihye Bae, Jihyeon Min, and Woojun Park***

Laboratory of Molecular Environmental Microbiology, Department of Environmental Science and Ecological Engineering, Korea University, Seoul 02841, Republic of Korea

**Keywords:** Gram-negative bacteria; cell wall; peptidoglycan; muropeptide transport; protein-protein interaction; bacterial metabolism

**^*^Corresponding author:** Dr. Woojun Park, Department of Environmental Science and Ecological Engineering, Korea University, Seoul, Republic of Korea, 02841

**E-mail:** wpark@korea.ac.kr

**Fax:** +82-2-953-0737

**Phone:** +82-2-3290-3067

**Supplementary Figure**

**Figure S1. Construction and PCR verification of deletion mutants in *A. baumannii* ATCC 17978.** Top, schematic of the CRISPR-Cas9 workflow used to generate gene deletions. Bottom panels, colony PCR of independent clones using primers flanking each locus. Wild-type and deletion allele sizes are indicated next to each gel; red arrows mark the deletion-allele band and black arrows mark the wild-type band. Lane numbers denote individual colonies.
**Figure S2. Morphological restoration of the complemented strain demonstrated by SEM analysis.** Stationary-phase WT, Δ*ampG1* mutant cells, and the complemented strain Δ*ampG1*/pBBR1MCS-2::P*ampG1*-*ampG1*-3×FLAG were imaged under identical conditions. Δ*ampG1* mutant cells appear rounded and swollen, whereas complementation restores the rod-like morphology. Scale bars, 1 μm.

**Figure S3. Growth, antibiotic susceptibility, and envelope–stress readouts across strains.** (A) Batch growth curves of WT, Δ*ampG1*, Δ*ampG2*, and Δ*ampG1*Δ*ampG2* quantified as CFU mL⁻¹ over time. (B) MIC heatmap for AMP, AMX, CTX, MEM, and LEX comparing WT and deletion strains. Values are in μg mL⁻¹. (C) Lysozyme challenge across a concentration series. Heatmap shows final OD_600_ for each strain after exposure. (D) Fosfomycin MIC assay presented as an OD₆₀₀ heatmap for all strains. (E) EDTA sensitivity at 2 mM measured as endpoint OD_600_. Error bars indicate the dispersion among three independent biological replicates.

**Figure S4. Architecture of AmpG1 and the *Acinetobacter*-specific periplasmic domain.** (A) AlphaFold model of *A. baumannii* AmpG1 positioned in the inner membrane using PPM 2.0. The protein comprises 14 transmembrane helices forming a core bundle, with an Ig-like periplasmic domain (PPD) connected by a short linker. N- and C-termini are indicated. (B) Topology map generated with Protter together with the corresponding structure views below. Transmembrane segments 1–14 are numbered, and the large periplasmic loop corresponds to the PPD. The N-terminal bundle comprising TM1–TM6 and the C-terminal bundle comprising TM9–TM14 are shown in contrasting blue and pink, respectively. TM7–TM8, the bundle bridge, the short linker region, and the PPD are shown in distinct colors. The overall structural model and a periplasmic top view illustrate the relative arrangement of the two major transmembrane bundles. The PPD was omitted from the top view for clarity. (C) HMM schematic for the AmpG1 PPD showing that conserved matches were identified only within the *Acinetobacter* genus.

**Figure S5. LC-MS/MS analysis of PG proportion of WT and Δ*ampG1* mutant cells.** (A) The table lists the peak identifier, muropeptide annotation, structural description, retention time, observed m/z and charge state, and relative abundance of each muropeptide in two independent biological replicates. Rep. 1 and Rep. 2 indicate independent biological replicates, and ND indicates that no quantifiable peak was detected in the indicated sample. Relative abundance was calculated from the integrated peak area of each muropeptide relative to the summed peak areas of all listed muropeptides within the same replicate. The summary section shows the summed abundance of assigned muropeptides and the proportions classified as monomers, dimers, trimers, and tetramers, together with the proportions of crosslinked muropeptides and anhydroMurNAc-muropeptides calculated independently for each biological replicate. (B and C) Proportions of crosslinked muropeptides (B) and anhydroMurNAc-containing muropeptides (C) in WT and Δ*ampG1* mutant cells. Bars show mean ± SD, and individual points represent two independent biological replicates. Crosslinking proportions were comparable between WT and Δ*ampG1* mutant cells and fell within the replicate-to-replicate variation, indicating that loss of AmpG1 does not appreciably alter overall crosslinking.

**Figure S6. Bocillin-FL labeling of penicillin-binding proteins in WT and Δ*ampG1* mutant cells.** Left, in-gel fluorescence of Bocillin-FL–labeled membrane proteins from WT and Δ*ampG1* run with equal total protein per lane. Major bands include PBP1a, PBP1b, PBP2, a TPase band (~67 kDa), PBP6b, and PBP7/8, with the TPase band appearing stronger in Δ*ampG1*. Right, reference table listing the assigned PBPs and the TPase band with their catalytic classes and approximate molecular masses used for band identification.

**Figure S7. SDS-PAGE analysis of purified SltΔ*SP* and AmpG1^PPD^ proteins.** (A) Sequential imidazole elution fractions (lanes 1–6) from Ni-NTA affinity purification of His6-tagged Slt lacking the signal peptide (Slt^ΔSP^), expressed in *E. coli* BL21 (DE3) carrying pET-28b(+)::*slt^Δsp^* and resolved on an 8% SDS-PAGE gel. A dominant band corresponding to Slt^ΔSP^ is observed at approximately 74 kDa. (B) Sequential imidazole elution fractions (lanes 1–4) from Ni-NTA affinity purification of His6-tagged AmpG1^PPD^, expressed in *E. coli* BL21 (DE3) carrying pET-28b(+)::*ampG1^PPD^* and resolved on a 12% SDS-PAGE gel, showing a major band at approximately 25.6 kDa. Lanes labeled C in panel B indicate whole-cell lysates. Fractions corresponding to lanes 1–4 were used for the MST and ITC binding assays for both Slt^ΔSP^ and AmpG1^PPD^.

**Figure S8. Isothermal titration calorimetry of the AmpG1^PPD^–Slt interaction.**
(A) Raw ITC thermogram for the titration of AmpG1^PPD^ into Slt, recorded at 25ºC. Each peak corresponds to the heat change following a single injection, and the upward (positive) deflections indicate that the binding reaction is endothermic. (B) Corresponding binding isotherm obtained by integrating the peak areas in (A) and plotting the heat per mole of injectant against the molar ratio. The integrated heats remained positive throughout the titration and are consistent with an endothermic interaction between AmpG1 ^PPD^ and Slt, although the curve did not reach saturation and the thermodynamic parameters derived from the fit carry substantial uncertainty.

**Figure S9. Adenylate cyclase-based bacterial two-hybrid assay using *A. baumannii* PG-related membrane proteins.** AmpG1^PPD^ was paired with PBP3(*ftsI*), PBP1B (*mrcB*), or PBP5 (*dacC*), and co-transformants were incubated on X-gal/IPTG indicator plates at 30°C for 48 h. Blue coloration indicates protein-protein interaction. The known interacting pair pKT25::*phoR* and pUT18::*mgtC* was used as a positive control. No positive signal was observed for the AmpG1^PPD^.

**Figure S10. LC-MS/MS quantification of PG precursors and anhydromuropeptides in WT, Δ*ampG1*, Δ*slt*, and Δ*ampG1*Δ*slt* mutant cells.** The table lists muropeptide annotation, structural description, and observed *m/z* ([M+H]^+^) for each targeted species, together with its retention time and integrated peak area in each strain across two independent biological replicates. The targeted species were the cytosolic precursor UDP-MurNAc-pentapeptide (UDP-M5) and the periplasmic turnover products GlcNAc-1,6-anhMurNAc-tripeptide (M3N) and GlcNAc-1,6-anhMurNAc-tetrapeptide (M4N). Rep. 1 and Rep. 2 denote the independent biological replicates, and ND indicates that no quantifiable peak was detected in the indicated sample. These replicate-level values are the source data for the mean ± SD shown in Figure 4D.

**Figure S11. β-Lactam susceptibility, β-lactamase activity, and membrane permeability.** (A) MIC heatmap for ampicillin (AMP), amoxicillin (AMX), cefotaxime (CTX), and meropenem (MEM) comparing WT and Δ*ampG1* (μg/mL). (B) β-Lactamase activity measured by nitrocefin hydrolysis at 486 nm. Left and top, baseline activity for WT, Δ*ampG1*, and blank. Others, activity in the presence of 1/4 MIC of AMP, AMX, or CTX. (C) Membrane barrier measurements. Left, ethidium bromide uptake over time. Right, NPN uptake. Δ*ampG1* exhibits lower dye uptake than WT under matched conditions.

**Figure S12. Phylogenetic placement of AmpG1, AmpG2, and AmpG3 within the broader MFS/AmpG space.** Circular phylogeny of AmpG-like transporters and related proteins compiled from representative bacterial, archaeal, and metagenome sequences. Branch lengths reflect substitutions per site and the outer ring indicates protein length. The *Acinetobacter baumannii* AmpG clade is annotated with AmpG1, AmpG2, and AmpG3, and reference sequences include canonical AmpG from *Klebsiella pneumoniae*, an AmpG from a hydrothermal-vent metagenome, and distant MFS transporter lineages. Perimeter cartoons show representative structures for labeled nodes (AlphaFold models), including clades annotated as peptidases and endo-N-acetylmuramidases and an archaeal MFS-containing protein, highlighting separation of non-AmpG families from the AmpG group.
