# Supplementary material for: Peptidoglycan permease AmpG1 couples lytic transglycosylase activity to muropeptide import in Acinetobacter baumannii
Source: J Biol Chem. 2026 Jul 24;302(9):113362. doi: 10.1016/j.jbc.2026.113362 (PMC13507758; doi:10.1016/j.jbc.2026.113362)
Supplement: Supplementary file 2 [file mmc3.docx]

**Table legends**

**Table S1. *In vivo* crosslinking proteomics data**

This Excel file lists proteins detected by LC–MS/MS after in vivo chemical crosslinking, membrane solubilization, and anti-FLAG immunoprecipitation of AmpG1_3xFLAG_ with label-free quantification. Columns include protein identifiers and annotations, basic peptide evidence (e.g., PSMs, unique peptides, sequence coverage), and abundance values. Detection in this dataset indicates association with the AmpG1_3xFLAG_ pulldown under the tested conditions but does not alone demonstrate a direct physical interaction.
