# Supplementary material for: Peptidoglycan permease AmpG1 couples lytic transglycosylase activity to muropeptide import in Acinetobacter baumannii
Source: J Biol Chem. 2026 Jul 24;302(9):113362. doi: 10.1016/j.jbc.2026.113362 (PMC13507758; doi:10.1016/j.jbc.2026.113362)
Supplement: Supplementary file 3 [file mmc5.docx]

**Table legends**

**Table S2. Oligonucleotides used in this study**

The table lists the oligonucleotide names, nucleotide sequences in the 5′–3′ direction, target genes, and experimental purposes.
